# Community perceptions of childbearing and use of safer conception strategies among HIV-discordant couples in Kisumu, Kenya

**DOI:** 10.7448/IAS.18.1.19972

**Published:** 2015-06-12

**Authors:** Brooke T Breitnauer, Okeoma Mmeje, Betty Njoroge, Lynae A Darbes, Anna Leddy, Joelle Brown

**Affiliations:** 1 Department of Obstetrics and Gynecology, University of Colorado School of Medicine, Aurora, CO, USA; 2 Department of Obstetrics and Gynecology, University of Michigan Medical School and Department of Health Behavior and Health Education, University of Michigan School of Public Health, Ann Arbor, MI, USA; 3 Centre for Microbiology Research, Kenya Medical Research Institute, Nairobi, Kenya; 4 Center for AIDS Prevention Studies, University of California, San Francisco, CA, USA; 5 Department of Health, Behavior and Society, Johns Hopkins Bloomberg School of Public Health, Baltimore, MD, USA; 6 Department of Epidemiology and Biostatistics, and Obstetrics, Gynecology, and Reproductive Sciences, University of California, San Francisco, CA, USA

**Keywords:** HIV-discordant couples, HIV transmission, HIV prevention, pregnancy, childbearing, safer conception, community perceptions

## Abstract

**Introduction:**

Safer conception strategies (SCS) have the potential to decrease HIV transmission among HIV-discordant couples who desire children. Community perceptions of SCS may influence the scale-up and uptake of these services, but little is known about how communities will react to these strategies. Without community support for SCS, their success as an HIV prevention tool may be limited. The objective of this study is to characterize community perceptions of SCS for HIV-discordant couples in Kisumu, Kenya, to inform ongoing and future safer conception intervention studies in low-resource settings.

**Methods:**

We conducted six focus group discussions and 11 in-depth-interviews in Kisumu, Kenya, among a diverse group (*N*=59) of community members, including men, women, youth (age 19–25), community health workers and local leaders. An iterative qualitative analysis using a grounded theory approach was employed.

**Results and discussion:**

All participants emphasized the importance of childbearing in their society and the right to have children, regardless of an individual's HIV status. While most participants believed that HIV-discordant couples should be allowed to have children, they discussed several barriers to the uptake of SCS such as HIV-related stigma, fear of HIV transmission to the uninfected partner and child, fear of unfamiliar medical procedures and lack of information among community members and health care providers about HIV prevention interventions that allow safer conception. Access to information, community experiences with successful safer conception interventions, healthcare provider training, male engagement and community mobilization may help overcome these barriers. Though assisted reproduction strategies generated the most negative reactions from participants, our results suggest that with education and explanation of these services, participants express interest in these strategies and want them to be offered in their community.

**Conclusions:**

Many community members noted a need and desire for safer conception education and services in Kisumu. However, community barriers such as fear, stigma and lack of information should be addressed before safer conception interventions can be successfully implemented and delivered. Further research focused on community education, male engagement and healthcare provider training is a crucial next step in delivering safer conception in this region.

## Introduction

Among HIV-positive individuals of reproductive age, the desire to conceive is common [1–5]. However, when HIV-discordant couples attempt natural conception, they place themselves at risk of HIV transmission [2,6]. An estimated 30–50% of HIV-positive people in sub-Saharan Africa (SSA) are involved in stable discordant relationships [7–9], and around 44–60% of incident HIV infections in parts of SSA occur in married or cohabiting discordant couples [10]. Because the risk of transmission is estimated to be higher in discordant couples who conceive [11], it is crucial that HIV prevention interventions focus on safer conception strategies (SCS) to reduce incident cases of HIV.

The goal of safer conception is to help support a couple's right and desire to conceive while at the same time decreasing the risk of HIV transmission. The SCS employed depend on which partner is HIV positive. There are several main strategies: treatment as prevention (TasP) with antiretroviral therapy (ART) in the HIV-positive partner, pre-exposure prophylaxis (PrEP) in the HIV-negative partner, vaginal insemination, sperm washing and voluntary medical male circumcision. These strategies can be used in combination or alone, depending on the couples’ preferences and clinical scenario [6,12]. The uptake and utilization of SCS will differ according to the social and cultural context, and the availability of financial resources [13,14].

Previous research suggests that community involvement is crucial to uptake, acceptability and the ultimate success of HIV-prevention interventions [15–18]. Although guidelines for safer conception have been outlined [12,19–24], little research explains how these various strategies will be received in practice. Guidance on pre-conception care for HIV-discordant couples in Kenya has recently been developed, and it outlines various SCS for HIV-discordant couples who desire conception, including initiation of ART at any CD4 count, viral load monitoring and suppression, limiting condomless intercourse to the fertile period, sperm washing and artificial vaginal insemination [20]. A key knowledge gap exists on community perceptions of SCS for HIV-discordant couples. The goal of this qualitative study was to characterize community perceptions of SCS for HIV-discordant couples in an HIV-endemic setting in Kenya. Results of this study will help inform ongoing and future safer conception intervention studies in Kenya and potentially other settings in SSA.

## Methods

The study was conducted in the city of Kisumu, Kenya, between April and May 2014. Kisumu County has one of the highest HIV prevalence estimates in Kenya at 19.3%, compared with 6.0% nationally [9,25,26].

We conducted six focus group discussions (FGDs) (*n*=8 participants each) and 11 in-depth interviews (IDIs). The FGDs and IDIs were conducted in a complementary manner to gather a wide range of responses and ensure that perspectives shared in a group setting were also noted in individual discussions. “Community member” was defined as any person living in Kisumu and surrounding villages who was not: 1) a healthcare worker with background and training in safer conception; or 2) currently utilizing SCS.

We recruited community members from urban areas of the city of Kisumu by purposive and snowball sampling methods. Prior to recruitment, we mobilized a group of community leaders and held an informational session about our research on SCS. We asked these key informants to recruit FGD and IDI participants from their existing networks, who in turn identified subsequent participants. This strategy allowed us to recruit a diverse group of male and female community members across the ages from various occupations. Each FGD was composed of a particular subgroup within the community: members of the initial community meeting, men, women, youth (males and females aged 19–25), community health workers and local leaders. IDIs were conducted with six additional women and five additional men. Study participants received reimbursement for travel and their time. While all participants were asked if they were in or had been in an HIV-affected relationship, participants’ HIV status was not assessed to avoid unnecessary discomfort and anxiety, and because the discussion focused on general community attitudes, rather than personal attitudes.

All discussions were facilitated by a trained moderator in the preferred language of the participants (i.e. Kiswahili, Luo or English). A semi-structured guide, informed by the theory of planned behaviour [27], prompted discussion on attitudes towards HIV-discordant couples having children, perceptions of SCS, and suggestions for implementation of safer conception services in Kisumu. During the discussion, participants were provided with an educational brochure that described the various SCS in simple terms. All audio recordings were transcribed verbatim by three professional transcriptionists who were familiar with the vernacular used by the interviewees. Transcriptionists were given instruction on the specific purposes of the study. One member of the study staff reviewed randomly selected audio recordings and transcripts to ensure their quality and consistency; if discrepancies were noted, the original interviewer was asked to modify the transcript as necessary. Transcripts were translated into English and imported into Dedoose^®^ software for coding. We performed an iterative qualitative analysis, using grounded theory as a framework. Two researchers (BB, AL) used open and axial coding to generate a codebook based on words, and differences in coding were resolved through discussion until consensus was reached [28]. After all data were coded, the investigators used an inductive framework to analyze emerging themes [29,30]. Fifty per cent of transcripts were double coded for quality assurance of the data analysis [29,30].

Ethical approval was obtained from the University of California, San Francisco, and the Kenya Medical Research Institute. All participants provided written informed consent.

## Results and discussion

A total of 59 community members were enrolled (Table 1). Overall, 41% of participants were male and 59% were female. The median age was 35 years (IQR=25–47). About one-third (36%) reported currently or previously being in an HIV-affected relationship. On average, FGDs took 90 minutes and IDIs took 45 minutes.

**Table 1:** Demographic characteristics of participants (*N*=59) in focus group discussions and in-depth interviews

	Total (*N*=59)	Focus group discussions (*N*=48)	In-depth interviews (*N*=11)
	
Characteristic	*N* (%)	*N* (%)	*N* (%)
Age (years)			
19–25	16 (27.1)	15 (31.3)	1 (9.1)
26–35	14 (23.7)	5 (10.4)	9 (81.8)
36–45	14 (23.7)	13 (27.0)	1 (9.1)
46–67	15 (25.4)	15 (31.3)	0
Gender			
Female	35 (59.3)	29 (60.4)	6 (54.5)
Male	24 (40.7)	19 (39.6)	5 (45.5)
Number of living children			
0	15 (25.4)	12 (25.0)	3 (27.3)
1–2	13 (22.0)	8 (16.7)	5 (45.4)
3–4	14 (23.7)	12 (25.0)	2 (18.2)
> 4	16 (27.1)	15 (31.3)	1 (9.1)
Missing data	1 (1.7)	1 (2.0)	0
Currently in or previously in an HIV-affected relationship^a^			
Yes	21 (35.6)	17 (35.4)	4 (36.4)
No	38 (64.4)	31 (64.6)	7 (63.6)
Employed			
Yes	44 (74.6)	34 (70.8)	10 (90.9)
No	15 (25.4)	14 (29.2)	1 (9.1)
Member of community group			
Yes	49 (83.1)	39 (81.3)	10 (90.9)
No	10 (16.9)	9 (18.8)	1 (9.1)

a Defined as at least a three-month romantic relationship between discordant or HIV-concordant partners.

Three major overlapping constructs were explored: perceptions of HIV-discordant couples having children, and facilitators and barriers to uptake of SCS. There were no major differences in responses provided by men, women, those with or without children or in HIV-affected partnerships or in FGDs versus IDIs.

### Positive attitudes about childbearing among HIV-discordant couples

Our results shed light on the complexity of community beliefs surrounding childbearing among HIV-discordant couples in Kisumu, Kenya. Most participants expressed positive attitudes about HIV-discordant couples having children, explained by the importance of fertility in relationships and the belief that couples have a right to have children. A majority of participants discussed the importance of having children in the context of their romantic relationships, family relationships and relationships within their community. Because of the importance of fertility in this community, most participants believed that HIV-discordant couples should be allowed, and even encouraged, to have children (Table 2).

**Table 2:** Positive and negative attitudes about childbearing among HIV-discordant couples

Positive attitudes	Importance of fertility	“Those who are [HIV] affected should have children. Without the child, there is no happiness in the family. You will have a house and cars but without the child, you won't be happy … I therefore believe these people should have children.” [Female, age 24, HIV-affected relationship, community leader FGD]
	Relationship strengthening	“It [safer conception strategies] will add a positive impact in that it will promote marriages. They will tend to have children who are free from HIV. Secondly, it will promote faithfulness.” [Female, age 23, not in HIV-affected relationship, youth FGD]
	Right to have children	“For those who have the information they will accept that it's their right as human beings to give birth.” [Male, age 19, HIV-affected relationship, community leader FGD]
Negative attitudes	Fear of transmission	“I think that the perceptions people have out here is that HIV-infected persons should not have children. One is because of the fear that the child they are going to have is going to be infected, so the major fear is transmission to the child and I think that has held them back from having children.” [Male, age 30, not in HIV-affected relationship, IDI]
	Stigma against HIV	“However, there are people who don't view those who are HIV positive as human beings. When a woman is pregnant and positive at the same time, people will say that the woman is pregnant and sick at the same time. Why must she bother herself with getting children?” [Female, age 48, HIV-affected relationship, community leader FGD]
	Lack of information	“I would also be happy if this information reaches people in the community. Once the information reaches the village, they will have the knowledge on HIV/AIDS … This [discrimination] is happening because people lack information. Things will change if you pass the right information to these people.” [Female, age 48, not in HIV-affected relationship, women FGD]

Participants explained that because fertility is critically important in this society, couples who do not have children might face problems in their marriage. Many participants talked about HIV-discordant couples experiencing infidelity or relationships ending because they could not conceive without putting the negative partner or child at risk. These participants explained that SCS could allow HIV-discordant couples to have children safely, ultimately strengthening the relationship and encouraging them to stay together. Balancing the potential to have a child with the very real threat of HIV transmission is a difficult task for HIV-discordant couples, and SCS may help to resolve this conflict [31].

Many participants expressed a belief that all couples, regardless of HIV status, had a right to have children. Often in contrast to those who lacked information and/or expressed fear of and stigma against HIV-positive individuals, these participants believed that reproduction was a basic human right. Our findings support prior studies showing the importance of childbearing in family relationships in SSA, regardless of HIV status [1–5,32].

### Negative attitudes about childbearing among HIV-discordant couples

Though many participants believed that HIV-discordant couples should be allowed to have children, they explained why others in their community might not agree. Negative perceptions about childbearing among HIV-discordant couples exist as a result of the fear of transmitting HIV to the partner and the child, general stigma against HIV and a lack of information about HIV prevention and SCS (Table 2).

Participants explained that community members fear that an HIV-positive woman would become very ill during pregnancy, that the child would become an orphan and that the community would have to assume responsibility of raising the child. In addition, many participants explained a common misperception in their community that HIV-positive women can only give birth to HIV-positive children. Those with prior knowledge of existing strategies to prevent mother-to-child transmission thought otherwise.

Many participants noted the prevalence of HIV-related stigma in their community. HIV-positive individuals can be discriminated against, viewed as outcasts or sinners. Several participants said that their community members believe that HIV is an abomination, that HIV-positive people will die soon and that they are even afraid to touch someone with the virus.

One of the main causes of these negative perceptions about childbearing among HIV-discordant couples was thought to be lack of information about HIV prevention and SCS.

### Barriers to the uptake of SCS

Participants discussed a number of barriers to the uptake of SCS. Major barriers include negative perceptions of these services, fear of HIV transmission, fear of unfamiliar medical procedures, HIV stigma, lack of information about the strategies and other socio-economic barriers (Table 3).

**Table 3:** Barriers and facilitators to uptake of safer conception

Barriers	Negative perceptions	“Caution will be thrown in the wind because the drug is there, eventually they might fail to get the drug and get the disease.” [Female, age 28, not in HIV-affected relationship, IDI]
	Fear of HIV transmission	“Some people are positive but desire to have children. There are those who are positive but live in fear. They fear that they will give birth to a HIV positive child.” [Female, age 37, HIV-affected relationship, women FGD]
	Fear of unfamiliar procedures	“Such a thing [non-intercourse vaginal insemination] has never happened in our community. I don't think it can happen. It is good. However, it is meant for other people-not in my community. You cannot suck the sperms and later insert in the vagina … Such things will scare the hell out of our community members.” [Female, age 47, not in HIV-affected relationship, women FGD]
	Stigma against HIV	“Number one [barrier] is stigma, the fear that people might have approaching the health practitioners or discussions that might lead to couples reaching a consensus to that kind of a plan. So stigma plays a big role in the whole thing.” [Male, age 30, not in HIV-affected relationship, IDI]
	Lack of SCS information	“It is true that the information that we lack is the source of our problem because we do not know what to do. We don't have an idea of what is to be done. And that is what is bringing to us a lot of problems.” [Male, age 24, HIV-affected relationship, men FGD]
	Financial barriers	“Key number one is the cost implications. If there will be costs involved in it … Majority of people like free things, if the services can be offered for free then they will come for them.” [Male, 30, HIV-affected relationship, IDI]
	Barriers to vaginal insemination and sperm washing	“In the Bible, it is abomination when you are having sex with a woman … even without having condoms and then you want to release outside … NO! According to the Bible, according to the Luo culture, you have to release inside … To take the sperm of a Luo man … I don't think that will happen.” [Male, age 51, not in HIV-affected relationship, initial community leader FGD]
	Lack of male involvement	“I think it is tricky here because if you were to use health facilities, most men will not go to these health facilities and especially the cases whereby the man is the one who is negative. There [that] is a problem.” [Male, age 50, not in HIV-affected relationship, initial community leader FGD]
Facilitators	Education	“The discussion has been fruitful to me. At least I have learnt things which are new and I didn't know. I have also got rid of some negative perceptions that I had. I am now clear on that. On that note, I think I am now better placed to do the dissemination of this information to the community.” [Female, age 24, FGD, not in HIV-affected relationship, youth FGD]
	Comparison to previous HIV-prevention interventions	“I think let's be realistic. Things have happened and the world is evolving. When the condom concept was brought into the community, there was quite a lot of resistance. Especially from the religious leaders … And they were actually preaching against it within the institutions. What is happening now about use of condoms? They are selling it and it is being used like a hot cake.” [Male, age 44, HIV-affected relationship, initial community leader FGD]
	Success stories and testimonials	“But as Luos say, people must always come to witness for themselves. I think they can easily encouraged if they see those who are in HIV-affected relationships getting healthy children. They will be encouraged and anyone who is positive will try their best to succeed in getting a child.” [Male, age 23, not in HIV-affected relationship, youth FGD]
	Community mobilization	“So, we should have such discussions everywhere in the community so that we all get the right information. We need the community members to have hope in life.” [Female, 48, not in HIV-affected relationship, community leader FGD]

HIV-related stigma and fear of knowing and disclosing one's HIV status was also noted as a major barrier. Addressing stigma and fear in this context will be critical as both stigma and fear can prevent HIV-affected patients from seeking care, and can prevent open conversations with healthcare providers [33,34].

Lack of knowledge about SCS was perceived as one of the most important barriers. Without widespread education on SCS in the community, many participants thought that the provision of SCS would not succeed. Efforts to educate the wider community are necessary and appropriate given that safer conception is relevant to both HIV-positive and negative individuals. Widespread education efforts may simultaneously help to reduce stigma by providing information to a wide range of community members, instead of targeting only HIV-positive people [35].

Participants discussed a number of financial, cultural and religious barriers to uptake of certain safer conception services. While some indicated that they would be willing to pay for services, many expressed concern that if the services were expensive, the majority of their community members would be unable to pay. Participants preferred a wide range of strategies, and no one subgroup came to a consensus on which strategy their community would prefer. Assisted reproduction strategies such as sperm washing and vaginal insemination generated the most negative reactions from participants, in comparison to other strategies such as TasP and PrEP. Several participants doubted that sperm washing and vaginal insemination would be acceptable in their community because of fears that the sperm might be used somewhere else, or fears that they would be seen as sinners. However, our results suggest that with education and explanation of these services, participants express interest in these strategies and want them to be offered in their community [5].

Another important barrier to delivering information through clinic settings is that men do not often visit the clinic or hospital. Though the community emphasized that men play an important role in reproductive decisions, it can be difficult to get men to come to the clinic for education. Efforts to engage men through community outreach efforts may improve knowledge and engagement among men in safer conception. Engagement of male partners in reproductive health programs in the region has proven to be integral to programs’ success [36,37].

### Facilitators to the uptake of SCS

Most participants said that providing education about HIV prevention and SCS will be critical in facilitating the uptake of these services. Several participants who came into the discussion with negative attitudes towards SCS changed their view once they received information on the topic (Table 3).

Several participants drew on experience from previously successful harm reduction interventions such as use of condoms, prevention of mother-to-child transmission [38], and voluntary medical male circumcision [39] to show why they thought their community members’ perceptions and uptake of SCS might improve with education.

Participants stated that local examples of success stories would help their community. Testimonials are an important way of sharing information among Luos, the largest ethnic group in western Kenya [40]. Once participants understood that HIV-discordant couples could have HIV-negative children and could reduce the risk of transmitting the HIV virus to the negative partner, they stated that they would be more likely to encourage others to take up these services. Testimonials from people who have had success with safer conception can be a powerful way of educating and encouraging other community members.

Participants believed the most important facilitators to the uptake of safer conception would be community education and mobilization with targeted male involvement. While participants thought it would be crucial for the information and services to be available in health facilities and to train healthcare providers on the information, they stressed the importance of wider community involvement. Many participants called for educating the wider community through various mechanisms, including radio, media, “baraza” (public meetings), roadshows and group forums.

### Strengths and limitations

Our study adds a unique perspective to the existing literature on safer conception by addressing community perspectives outside of the clinical setting. One strength of our study was the inclusion of both FGDs and IDIs, which generated similar responses and consistent attitudes amongst community members, despite demographic differences (age, gender, having children or not, HIV-affected relationship or not). Another strength includes the diversity of participants (age, gender, role in community, experience with HIV-affected relationships).

Limitations of this study include that it may not be representative of the entire population of Kenya, given the purposive and snowball sampling methods that were used. The findings may not be representative of regions in Kenya where HIV prevalence is lower; in addition, Kisumu is a research-rich environment and it is possible that there is a higher level of HIV-related knowledge in the area. Some participants may not have fully disclosed their opinions, as the topics of HIV status, discordant relationships and reproductive health can be sensitive.

## Conclusions

We found that many community members in Kisumu believe HIV-discordant couples should be allowed to safely conceive, and their communities desire information on safer conception and access to these services. However, certain barriers in the community such as fear of HIV transmission, stigma and lack of education must be addressed as SCS are integrated into HIV care and prevention services. Further research focused on community education, male engagement, and healthcare provider training is a crucial next step in delivering SCS in Kenya and other high HIV prevalent areas of SSA.
